# Age-adjusted Charlson comorbidity index predicts postoperative mortality in elderly patients with hip fracture: A prospective cohort

**DOI:** 10.3389/fmed.2023.1066145

**Published:** 2023-03-07

**Authors:** Dan-Long Zhang, Yu-Xuan Cong, Yan Zhuang, Xin Xu, Bin-Fei Zhang

**Affiliations:** ^1^Department of Trauma and Orthopedic Surgery, Honghui Hospital, Xi’an Jiaotong University, Xi’an, Shaanxi, China; ^2^Department of Joint Surgery, Honghui Hospital, Xi’an Jiaotong University, Xi’an, Shaanxi, China

**Keywords:** age-adjusted Charlson comorbidity index, mortality, elderly, hip fracture, Cox regression

## Abstract

**Background:**

This study aimed to evaluate the clinical association between the age-adjusted Charlson comorbidity index (aCCI) and postoperative mortality in elderly patients.

**Materials and methods:**

Elderly patients with hip fractures were screened from January 2015 to September 2019. After demographic and clinical characteristics were collected, linear and non-linear multivariate Cox regression models were used to identify the association between the aCCI and mortality. All analyses were performed using EmpowerStats and R software.

**Results:**

A total of 2,657 patients were included in the study, and the mean follow-up duration was of 38.97 months. The mean aCCI score was 4.24 ± 1.09, and 977 (34.14%) died of all-cause mortality. The fully-adjusted linear multivariate Cox regression models showed the aCCI to be associated with mortality [hazard ratio (HR) = 1.31, 95% confidence interval (CI):1.21–1.41, *P* < 0.0001]. Patients in Q2 showed greater mortality (HR = 1.60, 95% CI: 1.23–2.09; *P* = 0.0005) than those in Q1; patients in Q3 showed greater mortality (HR = 2.18, 95% CI: 1.66–2.87; *P* < 0.001) than those in Q1. In addition, the *P*-value for the trend also showed a linear association in the three models (*P* < 0.0001). In the sensitivity analysis, propensity score matching was used, and the results were stable.

**Conclusion:**

The mortality risk of hip fractures increased by 31% when the aCCI increased by one unit. aCCI score was shown to be a good predictor of three-year mortality following hip fracture.

**Clinical trial registration:**

http://www.chictr.org.cn/showproj.aspx?proj=152919, identifier ChiCTR2200057323.

## 1. Introduction

Geriatric hip fracture is a common complication of osteoporosis and a major problem worldwide. The total number of cases is expected to rise to approximately 2.6 million by 2025, with an increase to 7.3-21.3 million by 2050 (1, 2). The reported 1-year mortality rate is 22% (3). Therefore, the apparent consensus among researchers and surgeons is that as life expectancy improves and the overall age of the population increases, the associated burden on health services will rise in conjunction with the projected increase in the elderly population (4–7).

Many factors can predict mortality in patients with geriatric hip fractures including age, serum albumin, sodium, hemoglobin, arrhythmia, pneumonia, and heart failure (8, 9). A systematic review of preoperative predictors of mortality identified strong evidence for 12 predictors, including advanced age, male sex, poor preoperative ambulation status, higher American Society of Anesthesiologists (ASA) status, dementia, diabetes, cancer, cardiac disease, and multiple comorbidities (10).

Although there has been extensive focus and research on the predictors of post-hip fracture mortality, research regarding the use of risk prediction models is relatively limited. Previous studies have shown the Charlson comorbidity index (CCI) to predict postoperative complications in patients with surgically treated hip fractures (11) as well as mortality at 1-year follow-up (12). As patient age was subsequently determined to be correlated with prognosis (8, 9), the age-adjusted Charlson comorbidity index (aCCI), a modified version of the CCI, was introduced into clinical practice in 1994. The aCCI incorporates age as a correction variable of the final score by adding 1 point for every decade over 40 years of age (13), therefore it is especially suitable for geriatric patient populations. Even though the Elixhauser comorbidity index (ECI) has been used to predict mortality (14), it was reported that CCI provided a better prediction for in-hospital mortality than the ECI (15). However, the role of comorbidities in hip fractures has not yet been thoroughly evaluated. A better understanding of comorbidities can promote recognition of their prognostic implications for hip fractures. Moreover, whether the aCCI shows predictive performance in geriatric patients with hip fractures requires further verification. Thus, the purpose of this study was to use aCCI scores to predict long-term mortality and, consequently, help reduce post-hip fracture mortality. We hypothesized that a higher aCCI score would be associated with higher postoperative mortality and that aCCI score at admission could predict prognosis.

## 2. Materials and methods

### 2.1. Study design

This prospective cohort study recruited elderly adults who received treatment for hip fractures at the largest trauma center in Xi’an, China, from 1 January 2015, to 30 September 2019.

The ethics committee of the Xi’an Honghui Hospital approved this prospective study (No. 202201009). The requirement for informed consent was waived as patient identity remained anonymous and because of the observational nature of the study, as reported elsewhere (16, 17). All human procedures were performed in accordance with the 1964 Declaration of Helsinki and its later amendments. The STROCSS 2021 guidelines were followed (18).

### 2.2. Participants

The demographic and clinical data of the reviewed patients were obtained from their original medical records. The inclusion criteria were as follows: patients who had (1) age ≥65 years; (2) diagnosis of the femoral neck, intertrochanteric, or subtrochanteric fracture by X-ray or computed tomography; (3) surgical or conservative treatment in the hospital; (4) availability of clinical data in the hospital; and (5) the ability to be contacted by telephone. We excluded patients who could not be successfully contacted.

### 2.3. Hospital treatment

After admission, patients underwent blood tests and ultrasonography to prepare for surgery. Closed/open reduction and internal fixation of proximal femoral nail anti-rotation are often chosen for intertrochanteric fractures, whereas femoral neck fractures are often treated with hemiarthroplasty or total hip arthroplasty, depending on patient age. In this study, conservative treatment was chosen for some patients owing to the risks associated with surgery. For all surgical patients, prophylaxis for deep vein thrombosis was initiated on admission. At discharge, patients were asked to return for assessment of fracture union or function monthly.

### 2.4. Follow-up

Patients’ family members were contacted by telephone from January 2022 to March 2022 to record data including survival or death, survival time, and activities of daily living after discharge. Telephone follow-up was conducted by two medical professionals with one year of experience after two weeks of training. For patients who could not be contacted by telephone in the first round, two additional attempts were made. When the patients’ family members were unreachable for the third time, treatment was stopped and the patient was recorded as lost to follow-up.

### 2.5. Endpoint events

The singular endpoint event in this study was all-cause mortality after treatment. We defined all-cause mortality as death reported by the patients’ family members.

### 2.6. Variables

The following variables were collected: age, sex, occupation, history of allergy, injury mechanism, fracture classification, hypertension, diabetes, coronary heart disease, arrhythmia, hemorrhagic stroke, ischemic stroke, cancer, multiple injuries, dementia, chronic obstructive pulmonary disease, hepatitis, gastritis, aCCI score, time from injury to admission, time from admission to operation, operation time, blood loss, infusion, transfusion, treatment strategy, length of hospital stay, and follow-up. Occupations included retirement, farming, and others; injury mechanisms included falls, accidents, and other unintentional causes. each comorbidity. We calculated the aCCI score by counting each comorbidity and adjusting age.

### 2.7. Statistics analysis

Data are presented as mean ± standard deviation (SD) (Gaussian distribution) or median (range) (skewed distribution) for continuous variables, and as numbers and percentages for categorical variables. The χ^2^ (categorical variables), one-way ANOVA (normal distribution), or Kruskal–Wallis H test (skewed distribution) were used to detect the differences among different aCCI scores. We divided the patients into Q1–Q3 subgroups (tertiles) according to aCCI scores distribution. Q1–Q3 were in ascending order of CCI scores. To examine the association between aCCI and mortality, three distinct models using univariate and multivariate Cox proportional hazards regression were constructed: a non-adjusted model (no covariates were adjusted), minimally adjusted model (only sociodemographic variables were adjusted), and fully adjusted model. Effect sizes with 95% confidence intervals were recorded. To account for the non-linear relationship between aCCI and mortality, a Cox proportional hazards regression model with cubic spline functions and smooth curve fitting (penalized spline method) was used to address non-linearity. In addition, a two-piecewise Cox proportional hazards regression model was used to further explain non-linearity.

To test the robustness of our results, sensitivity analysis was performed. aCCI score was converted into a categorical variable according to tertiles and *P*-value was calculated for the trend to verify the results of aCCI score as a continuous variable and to examine the possibility of non-linearity. In addition, propensity score matching (PSM) was used to compare the matched groups.

Modeling was performed using statistical software packages R (The R Foundation)^1^ and EmpowerStats (X&Y Solutions Inc., Boston, MA, USA).^2^ Hazard ratios (HR) and 95% confidence intervals (CI) were calculated. Statistical significance was set at *P* < 0.05 (two-sided).

## 3. Results

### 3.1. Patient characteristics

This study enrolled 3,242 consecutive participants with hip fractures from January 2015 to September 2019; of them, 585 patients (18%) were lost to follow-up. Ultimately, 2,657 participants were included in this study. The mean follow-up duration was 38.9 months, mean aCCI score was 4.24 ± 1.09, and mortality rate was 34.14%. Based on the distribution, aCCI was divided into three groups (Q1–Q3). Demographic and clinical characteristics including comorbidities, factors associated with injuries, and treatment strategies are shown in Table 1.

**TABLE 1:** The demographic and clinical characteristics (*N* = 2,657).

aCCI group	Q1 (*n* = 624)	Q2 (*n* = 1083)	Q3 (*n* = 950)	*P*-value	*P*-value*
aCCI	2.82 ± 0.39 [2,3]	4.00 ± 0.00 [4]	5.40 ± 0.66 [5,9]	<0.001	<0.001
Age (year)	72.88 ± 4.32	80.89 ± 6.39	82.58 ± 5.37	<0.001	<0.001
Sex				0.51	–
Male	214 (34.29%)	342 (31.58%)	312 (32.84%)		
Female	410 (65.71%)	741 (68.42%)	638 (67.16%)		
Occupation				<0.001	–
Retirement	307 (49.20%)	633 (58.45%)	599 (63.05%)		
Farmer	210 (33.65%)	249 (22.99%)	176 (18.53%)		
Other	107 (17.15%)	201 (18.56%)	175 (18.42%)		
History of allergy	21 (3.37%)	39 (3.60%)	46 (4.84%)	0.239	-
Injury mechanism				<0.001	–
Falling	589 (94.39%)	1052 (97.14%)	927 (97.58%)		
Accident	32 (5.13%)	21 (1.94%)	16 (1.68%)		
Other	3 (0.48%)	10 (0.92%)	7 (0.74%)		
Fracture classification				<0.001	–
Intertrochanteric fracture	416 (66.67%)	805 (74.33%)	719 (75.68%)		
Femoral neck fracture	183 (29.33%)	256 (23.64%)	210 (22.11%)		
Subtrochanteric fracture	25 (4.01%)	22 (2.03%)	21 (2.21%)		
Hypertension	224 (35.90%)	515 (47.55%)	555 (58.42%)	<0.001	–
Diabetes	29 (4.65%)	158 (14.59%)	341 (35.89%)	<0.001	–
CHD	279 (44.71%)	538 (49.68%)	595 (62.63%)	<0.001	–
Arrhythmia	147 (23.56%)	390 (36.01%)	357 (37.58%)	<0.001	–
Hemorrhagic stroke	6 (0.96%)	27 (2.49%)	26 (2.74%)	0.048	–
Ischemic stroke	27 (4.33%)	183 (16.90%)	561 (59.05%)	<0.001	–
Cancer	1 (0.16%)	14 (1.29%)	63 (6.63%)	<0.001	–
Multiple injuries	39 (6.25%)	74 (6.83%)	79 (8.32%)	0.244	–
Dementia	1 (0.16%)	10 (0.92%)	96 (10.11%)	<0.001	–
COPD	4 (0.64%)	28 (2.59%)	143 (15.05%)	<0.001	–
Hepatitis	4 (0.64%)	19 (1.75%)	62 (6.53%)	<0.001	–
Gastritis	2 (0.32%)	9 (0.83%)	35 (3.68%)	<0.001	–
Treatment strategy				<0.001	–
Conservation	18 (2.88%)	75 (6.93%)	143 (15.05%)		
ORIF	432 (69.23%)	752 (69.44%)	600 (63.16%)		
HA	145 (23.24%)	251 (23.18%)	206 (21.68%)		
THA	29 (4.65%)	5 (0.46%)	1 (0.11%)		
Time to admission (h)	69.76 ± 282.49	67.03 ± 146.42	104.74 ± 304.22	0.001	<0.001
Stay in hospital (day)	8.52 ± 3.35	8.77 ± 3.51	9.43 ± 4.16	<0.001	<0.001
Time to operation (day)	4.19 ± 2.70	4.21 ± 2.47	4.52 ± 2.62	0.019	0.007
Operation time (min)	96.54 ± 39.41	92.70 ± 35.95	94.25 ± 36.99	0.133	0.09
Blood loss (ml)	247.91 ± 176.60	239.55 ± 142.60	252.06 ± 177.16	0.264	0.472
Infusion (ml)	1623.73 ± 417.78	1537.43 ± 371.85	1540.75 ± 375.11	<0.001	<0.001
Transfusion (U)	1.02 ± 1.30	1.16 ± 1.24	1.25 ± 1.30	0.004	<0.001
Follow-up (months)	46.08 ± 17.95	39.21 ± 19.57	34.04 ± 19.60	<0.001	<0.001
Mortality	94 (15.06%)	376 (34.72%)	437 (46.00%)	<0.001	–

Results in the table: Mean + SD/*N*(%). *P*-value*: For continuous variables, we used the Kruskal–Wallis rank-sum test and Fisher’s exact probability test for count variables with a theoretical number < 10.

### 3.2. Univariate analysis of variables and mortality

To identify adjusted factors and the relationship between variables and mortality, we performed a univariate analysis as shown in Table 2. According to the set criteria of *P* < 0.1, the following variables were considered in the multivariate Cox regression: age, sex, occupation, injury mechanism, fracture classification, hospital stay, time to admission, time to operation, treatment strategy, operation time, and infusion.

**TABLE 2:** Effects of risk factors on mortality by univariate analysis.

	Statistics	HR (95% CI)	*P*-value
aCCI	4.22 ± 1.08	1.51 (1.43, 1.60)	<0.0001
Age (year)	79.61 ± 6.76	1.08 (1.07, 1.09)	<0.0001
Sex			
Male	868 (32.67%)	1	
Female	1789 (67.33%)	0.74 (0.65, 0.85)	<0.0001
Occupation			
Retirement	1539 (57.92%)	1	
Farmer	635 (23.90%)	0.90 (0.77, 1.06)	0.1943
Other	483 (18.18%)	0.84 (0.70, 1.00)	0.0511
History of allergy			
No	2551 (96.01%)	1	
Yes	106 (3.99%)	0.86 (0.59, 1.23)	0.4029
Injury mechanism			
Falling	2568 (96.65%)	1	
Accident	69 (2.60%)	0.25 (0.12, 0.52)	0.0002
Other	20 (0.75%)	1.59 (0.85, 2.97)	0.1449
Fracture classification			
Intertrochanteric fracture	1940 (73.01%)	1	
Femoral neck fracture	649 (24.43%)	0.86 (0.72, 1.02)	0.0811
Subtrochanteric fracture	68 (2.56%)	0.76 (0.49, 1.17)	0.2109
Stay in hospital (day)	8.95 ± 3.74	1.03 (1.01, 1.04)	0.0021
Time to admission (h)	81.15 ± 246.64	1.00 (1.00, 1.00)	0.0282
Time to operation (day)	4.31 ± 2.58	1.02 (1.00, 1.05)	0.0783
Treatment strategy			
Conservation	236 (8.88%)	1	
ORIF	1784 (67.14%)	0.32 (0.27, 0.38)	<0.0001
HA	602 (22.66%)	0.34 (0.27, 0.42)	<0.0001
THA	35 (1.32%)	0.06 (0.02, 0.26)	0.0001
Operation time (min)	94.18 ± 37.20	1.00 (1.00, 1.00)	0.0559
Blood loss (ml)	245.80 ± 163.48	1.00 (1.00, 1.00)	0.3248
Infusion (ml)	1560.27 ± 386.58	1.00 (1.00, 1.00)	0.0001
Transfusion (U)	1.16 ± 1.28	1.04 (0.98, 1.10)	0.199

### 3.3. The multivariate analysis between aCCI and mortality

**FIGURE 1:**
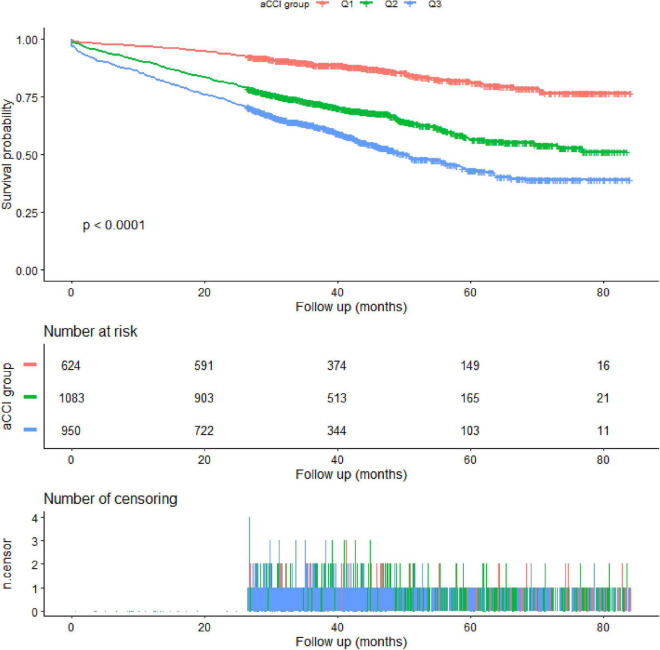
The Kaplan–Meier survival curve.

As shown in Table 3, three models were used to demonstrate the association between aCCI score and mortality. When aCCI score was a continuous variable, stable linear regression was observed. The fully adjusted model showed that the mortality risk increased by 31% (HR = 1.31, 95% CI: 1.21-1.41, *P* < 0.0001) when the aCCI increased by one unit. When the aCCI was changed to a categorical variable, we found statistical differences in the Q2 and Q3 groups compared with the Q1 group in all three models. In addition, the *P*-value for the trend also showed a linear association in the three models (*P* < 0.0001). The Kaplan–Meier survival curves are shown in Figure 1.

**TABLE 3:** Multivariate results by Cox regression.

Exposure	Non-adjusted model	Minimally-adjusted model	Fully-adjusted model
aCCI	1.51 (1.43, 1.60) < 0.0001	1.33 (1.24, 1.42) < 0.0001	1.31 (1.21, 1.41) < 0.0001
aCCI group			
Q1	Ref	Ref	Ref
Q2	2.69 (2.14, 3.37) < 0.0001	1.78 (1.38, 2.28) < 0.0001	1.60 (1.23, 2.09) 0.0005
Q3	4.08 (3.27, 5.11) < 0.0001	2.51 (1.95, 3.23) < 0.0001	2.18 (1.66, 2.87) < 0.0001
*P* for trend	<0.0001	<0.0001	<0.0001

Data in table: HR (95% CI) *P*-value. Outcome variable: Mortality. Exposed variables: aCCI. The minimally adjusted model was adjusted for age and sex. The fully adjusted model was adjusted for age, sex, occupation, injury mechanism, fracture classification, length of hospital stay, time to admission, time to surgery, treatment strategy, operation time, and infusion.

### 3.4. Analysis of threshold or saturation effect

Table 4 compares the two fitting models to explain the association between aCCI and mortality. Ultimately, we found that there was no threshold or saturation effect (*P* = 0.103).

**TABLE 4:** Non-linearity addressing of aCCI and mortality.

Outcome	HR (95% CI) *P*-value
Fitting model by stand linear regression	1.31 (1.21, 1.41) < 0.0001
Fitting model by two-piecewise linear regression	
Inflection point	4
<4	1.56 (1.24, 1.96) 0.0002
>4	1.26 (1.15, 1.38) <0.0001
*P*-value for the log-likelihood ratio test	0.103

Adjusted strategy was the same as the fully-adjusted model in Table 3.

### 3.5. Propensity score matching (PSM)

**FIGURE 2:**
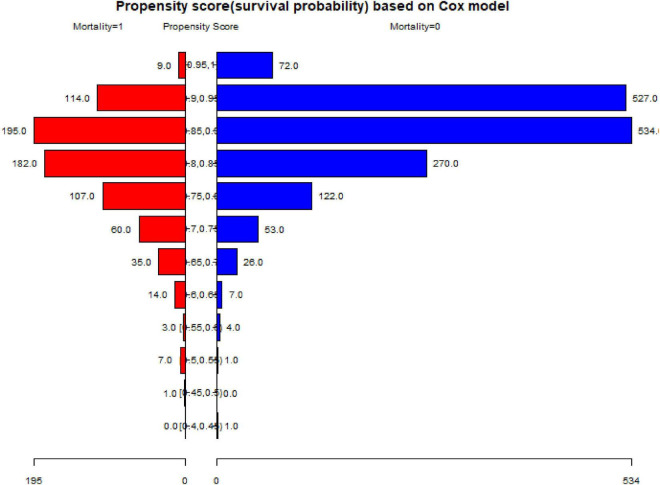
The PSM of two groups under propensity score based on the Cox model.

To test the robustness of our results, we performed a sensitivity analysis using PSM, as shown in Tables 5–7 and Figure 2. A total of 1,448 patients were successfully matched. Age, occupation, fracture classification, and treatment strategy did not match between the two groups. The results in the multivariate Cox regression under the PSM and PSM-adjusted models, were found to be stable.

**TABLE 5:** Propensity score parameter list.

The variables used in calculating the propensity score	Age, sex, occupation, injury mechanism, fracture classification, stay in the hospital, time to admission, time to operation, treatment strategy, operation time, and infusion
Propensity score algorithm	Cox regression model
C-statistical	0.6898
Matching method	Greedy matching within specified caliper distances
Metric Distances	0.05
Matching ratio	1:1
Use of replacement	With replacement
Matching sample size	No. of survival = 1: 724 cases No. of dead = 0: 724 cases Total 1,448 cases

**TABLE 6:** The balance test of PSM.

Variables	Mortality: Alive (724)	Mortality: Dead (724)	Standardized difference	*P*-value
	**Survival**	**Dead**		
Age (year)	83.43 ± 4.46	81.94 ± 6.34	0.2726	<0.0001*
Sex			0.0229	0.7032
Male	264 (36.5)	272 (37.6)		
Female	460 (63.5)	452 (62.4)		
Occupation				0.0441*
Retirement	471 (65.1)	433 (59.8)	0.1085	
Farmer	129 (17.8)	166 (22.9)	0.1271	
Other	124 (17.1)	125 (17.3)	0.0037	
Fracture classification				0.009*
Intertrochanteric fracture	515 (71.1)	560 (77.3)	0.1425	
Femoral neck fracture	199 (27.5)	150 (20.7)	0.1587	
Subtrochanteric fracture	10 (1.4)	14 (1.9)	0.0433	
Injury mechanism				0.1494
Falling	709 (97.9)	711 (98.2)	0.0201	
Accident	13 (1.8)	7 (1)	0.0711	
Other	2 (0.3)	6 (0.8)	0.0746	
Treatment strategy				0.0138*
ORIF	513 (70.9)	561 (77.5)	0.1519	
HA	207 (28.6)	161 (22.2)	0.1463	
THA	4 (0.6)	2 (0.3)	0.043	
aCCI				0.0343*
2	2 (0.3)	8 (1.1)	0.1002	
3	60 (8.3)	78 (10.8)	0.0847	
4	355 (49)	313 (43.2)	0.1166	
5	222 (30.7)	213 (29.4)	0.0271	
6	66 (9.1)	83 (11.5)	0.0773	
7	17 (2.3)	23 (3.2)	0.0506	
8	2 (0.3)	6 (0.8)	0.0746	
Stay in hospital (day)	8.91 ± 3.43	9.19 ± 3.63	0.0807	0.1251
Time to admission (h)	94.74 ± 309.46	85.24 ± 155.93	0.0387	0.4612
Time to operation (day)	4.42 ± 2.79	4.54 ± 2.88	0.04	0.447
Operation time (min)	90.56 ± 33.02	91.73 ± 33.14	0.0355	0.4996
Infusion (ml)	1484.81 ± 336.43	1505.03 ± 371.14	0.0571	0.2776

*Variables were not successfully matched.

**TABLE 7:** Multivariate results by Cox regression under the PSM model.

Exposure	PSM model	PSM-adjusted model
aCCI	1.10 (1.02, 1.19) 0.0189	1.19 (1.10, 1.29) < 0.0001

Data in table: HR (95% CI) *P*-value. Outcome variable: Mortality. Exposed variables: aCCI. Adjusted model for age, occupation, fracture classification, and treatment strategy.

## 4. Discussion

The results of this study demonstrated a linear association between aCCI score and mortality after geriatric hip fracture, with a 31% increase in mortality (HR = 1.31, 95% CI: 1.21–1.41; *P* < 0.0001) and a one-unit increase in aCCI. Patients in Q2 showed greater mortality (HR = 1.60, 95% CI: 1.23–2.09; *P* = 0.0005) than those in Q1, and patients in Q3 showed greater mortality (HR = 2.18, 95% CI: 1.66–2.87; *P* < 0.001) than those in Q1. Clinically, we suggest using the aCCI score at admission to predict prognosis in elderly patients with hip fracture. By improving the management of potentially controllable disorders, we can improve patient survival rates.

The aCCI is a significant predictor of survival after cancer (19), infective endocarditis (20), transcatheter mitral valve repair (21), and COVID-19 pneumonia (22). Presently, there are numerous studies on the association between CCI and mortality in patients with hip fracture, all having reported that high CCI scores were associated with mortality or considered high CCI score to be a risk factor (8, 12, 23–25). In one study, Pan et al. (8) reported that the risk variables for mortality after hip fracture surgery in geriatrics were age, albumin level, sodium level, hemoglobin level, and CCI score (HR = 1.38) in 45 patients (8, 12). Additionally, Hjelholt et al. (26) developed a user-friendly prediction tool for 1-year mortality in patients with hip fractures. The final model included nursing home residency, CCI score, cumulative ambulation score, body mass index, and age; had acceptable discrimination and calibration; and predicted one-year mortality risk ranging from 5 to 91% depending on the combination of predictors in the individual patient.

Age (27), sex (28), fracture classification (27), time to operation (29), treatment strategy (30), and hospital stay (31) were reported as risk factors in previous studies. In addition, we considered the adjusted the factor of *P* < 0.1 in the univariate analysis: occupation, injury mechanism, time to admission, operation time and infusion. Thus, we comprehensively considered the variables that needed to be adjusted. Age is a very important factor that should be considered in geriatric hip fracture and adjusted during data analyses. In a cross-sectional study by Padrón-Monedero et al. (32), the results showed an association between age and mortality following hip fracture after adjusting for numerous comorbidities. The aCCI considers age, whereas the CCI does not; aCCI, which considered old age a risk factor, was more accurate. In their study, Jiang et al. (33) retrospectively assessed the association between the aCCI and 5-year mortality in a surgically treated hip fracture population of 1,057 patients. The results demonstrated that patients with aCCI ≥ 6 had an increased 5-year mortality rate with an odds ratio of 13.6 compared to those with aCCI ≤ 3. Moreover, a study by Gatot et al. (34) concluded that an aCCI ≥ 6 could predict higher 90-day readmission rates, poor quality of life, and poor potential for functional recovery 1-year post-operation in patients with hip fracture. As it used a prospective observational design, this study provided stronger association, allowing for better interpretation of mortality. In the present study, the longest follow-up was 84.19 months with an average of 38.9 months, which was longer than the 1 year used in a study by Chen et al. (35) and Garabano et al. (24), as well as the 2 years used in a study by Cher et al. (25), and 37.2 months in a study by Pan et al. (8). The sample size in our study was also larger than those used in these previous studies (8, 24, 25, 33, 35). In addition, the results of the sensitivity analysis in the present study were stable.

Comorbidities are quite common in elderly patients with hip fractures (36, 37), and the assessment of prognosis after injury is usually insufficient. The ASA is a typically utilized tool (38, 39); however, Varady et al. (40) found that the aCCI was more accurate than the ASA score for 1-year mortality after hip fracture surgery. The ECI is another popular tool for predicting the prognosis of hip fractures; however, Tang et al. (15) found that aCCI provided a better prediction of in-hospital mortality than ECI among elderly patients. Moreover, these data validate that aCCI can be reliably performed in the International Classification of Diseases-10 era. Because of the objective nature of these indices, the aCCI may be a useful preoperative measure for surgeons to assess mortality in hip fracture patients and should likely be used for institutional orthopedic research involving outcomes at 90 days and beyond (40).

To the best of our knowledge, this prospective study had the largest sample size used to date to explain the linear association between aCCI score and mortality in geriatric hip fractures. Our findings provide new insights into the association between aCCI score and mortality and contribute to clinical evidence on the use of comorbidities at admission in predicting the prognosis of elderly patients with hip fractures. The relationship between aCCI score and mortality was shown to be linear rather than non-linear, and there was no threshold or saturation point. Therefore, mortality risk increased following the addition of the aCCI. The higher the aCCI score was, the poorer the result. Clinically, we suggest that surgeons calculate aCCI scores at admission and use it to predict prognosis in the median term.

This was a prospective study with a large sample size, and although the loss-to-follow-up rate was 18%, we found that the patients who were lost to follow-up were randomized, and most of the variables in Table 1 were comparable between the present and absent groups. Furthermore, we included patients who were admitted in September 2019. One important reason was to avoid the effect of COVID-19 (41, 42) and another was to ensure follow-up over 2 years. During the analysis, to explore the real relationship between the two factors, we not only carried out linear regression using various adjusted models but also changed the continuous variable of access to a categorical variable or performed a trend test for the result. In addition, we explored the association with the curve relationship and found no threshold or saturation effect, which supplemented the stability of the linear association. We also performed sensitivity analysis using PSM to test the robustness of our results; a total of 1448 patients were matched successfully and the results remained quite stable.

However, this study has some limitations. First, as it was a prospective study, loss to follow-up (18%) was inevitable. We attempted to reach the patients three times by phone to obtain outcome data. Second, the results only apply to patients aged ≥65 years and not to younger patients. Third, the samples of this study were from China; thus, the conclusions have certain regional and ethnic restrictions, and the inference points for other races should be redefined.

Clinically, we suggest that aCCI score at admission should be used to predict prognosis for elderly patients with hip fracture.

## Data availability statement

The original contributions presented in this study are included in the article/supplementary material, further inquiries can be directed to the corresponding authors.

## Ethics statement

The studies involving human participants were reviewed and approved by the Ethics Committee of the Honghui Hospital (No. 202201009). The patients/participants provided their written informed consent to participate in this study.

## Author contributions

XX and B-FZ conceived and designed the study. B-FZ, Y-XC, and YZ analyzed the data. D-LZ and B-FZ wrote the manuscript. All authors contributed to the article and approved the submitted version.

## Footnotes

1
http://www.R-project.org

2
http://www.empowerstats.com
